# Primary or secondary wound healing of the pin sites after removal of the external fixator: study protocol for a prospective, randomized controlled, monocenter trial

**DOI:** 10.1186/s13063-020-4087-8

**Published:** 2020-02-19

**Authors:** Franz Roth, Flavio Cagienard, Björn C. Link, Sandro Hodel, Dirk Lehnick, Reto Babst, Frank J. P. Beeres

**Affiliations:** 1 Lucerne Cantonal Hospital, Orthopedic and Trauma Surgery, Lucerne, Switzerland; 2 Clinical Trial Unit Central Switzerland and its Head of Biostatistics and Methodology, Lucerne, Switzerland

**Keywords:** Fixator external, Fixateur externe, External fixator, Wound healing, Fix-Ex, Pin site, Trauma, Infection, Pin-tract infection

## Abstract

**Background:**

Temporary fixation with an external fixator is used for numerous indications in orthopedic trauma surgery. It is unclear whether primary wound healing or secondary open-wound healing after removal of the external fixator should be advocated for the pin site. This study compares primary wound closure with secondary wound healing for the pin site. The primary aim is to compare pin-site infection rates. The secondary aim is to compare time to wound healing and esthetic outcome. The hypothesis was that primary wound closure does not lead to more infections than secondary wound healing.

**Methods and design:**

This is a prospective, randomized controlled, blinded, monocenter study based on a non-inferiority design. To obtain an equal patient population and groups, all pin-entry sites of the patients are treated alternately at the time of removal of the external fixator with primary wound closure and secondary wound healing. Patients are randomized according to whether the proximal pin-entry site is treated with wound closure or by secondary open-wound healing, from which the further sequence develops. The pre- and postoperative protocol is standardized for all pin-entry sites. A photo documentation of the pin-entry sites takes place 2 and 52 weeks postoperatively during the routine clinical follow-up visits. Further controls take place at 6, 12 and 26 weeks after pin removal.

The primary outcome was to demonstrate the non-inferiority of primary wound closure compared to secondary wound healing in terms of postoperative wound infections according to the Center of Disease Control and Prevention (CDC) definitions.

The secondary outcomes are time to complete wound healing (days) and esthetical outcome (subjective preference of patients and Vancouver Scar Scale score).

**Discussion:**

This study aims to answer how to deal with the pin site after removal of the external fixator. To date, no routine and generally accepted protocol exists for the management of pin sites after removal of the external fixator. This prospective, randomized controlled, blinded monocenter trial should determine whether primary wound closure or secondary wound healing should be advocated after removal of the external fixator.

**Trial registration:**

ClinicalTrials.gov, ID: NCT03842956. Registered retrospectively on 13 February 2019.

## Background

In orthopedic trauma surgery, the use of temporary external fixators is common [1–3]. Pin-entry site infections are frequently seen complications with infection rates up to 7.4% [4–7]. These infections can cause pain and discomfort to the patient and can lead to osteomyelitis. It is unclear whether primary wound closure or secondary open-wound healing after removal of the external fixator should be the standard of care for pin sites to achieve a lower infection rate and better esthetic outcome [1, 6, 8]. Despite it being one of the basic procedures in orthopedic trauma, the wide variety of ways of managing the pin site is underlined in a recent international survey [1]. To date, no routine and generally accepted protocol exists for the management of pin sites after removal of the external fixator [1]. The primary aim of this prospective, randomized controlled, blinded, monocenter trial is to evaluate whether primary wound closure or secondary wound healing is advocated after removal of the external fixator. The hypothesis was that primary wound closure does have a similar infection rate compared to open-wound healing according to the Center for Disease Control and Prevention (CDC) definitions [9].

The secondary aim is to investigate the time to complete wound healing, and the aesthetic outcome.

## Methods and design

### Study design

This prospective, randomized controlled, blinded, monocenter study, based on a non-inferiority study design, is enrolled in a Level-1 Trauma Centre in Central Switzerland. A total of 234 pin sites (± 70 patients) will be included. Ethical approval of this study was obtained from the Swiss Ethics Board with the project ID: 2018–01316 (Additional file 1).

### Patient population

**Fig. 1:**
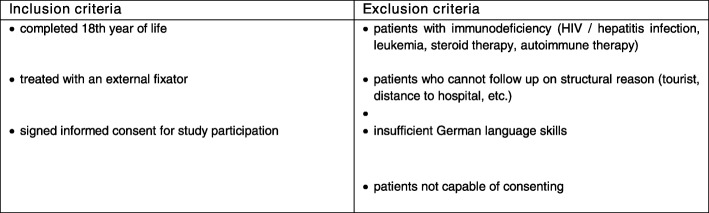
Inclusion and Exclucion criteria

All patients treated with a temporary external fixator are screened for eligibility. The inclusion and exclusion criteria are shown in Fig. 1. All pin sites except the pin sites at the calcaneus, due to the low mobilizability of the skin and thus a lack of tension-free wound closure, will be included. After obtaining both, written and oral informed consent, patients are included.

### Inclusion and exclusion criteria

The population includes all patients who are aged 18 years or older and who were treated with an external fixator in our hospital. Patients with immunodeficiency or patients who cannot follow up on structural reasons are excluded. Patients with a lack of knowledge of German or a lack of consent to study participation are also excluded. The inclusion and exclusion criteria are listed in Fig. 1.

### Randomization process

To obtain an equal patient population or groups regarding preexisting conditions, health status and trauma condition, all pin sites of the patients are treated at the time of removal of the external fixator alternately with a primary wound closure and by secondary wound healing. If a patient has consented to the study, the patient will be allocated to group A or group B by using a computerized randomization. Patients assigned in group A, the proximal pin site will be closed by a single-button suturing according to the Allgoewer technique. In the patients of group B, the proximal pin site is treated by secondary open-wound healing.

### Intervention

The pre- and postoperative protocol is standardized for all pin sites, including preoperative antibiotic prophylaxis with a single preoperative dose of cefazolin 2 g given intravenously (i.v.) 30–60 min prior to surgery. The pin sites assigned to the intervention group will be treated according to our current standard protocol, which implies that they will be closed by the single-button technique. The control group is simultaneously subjected to secondary wound healing without wound closure.

### Postoperative management

No routine postoperative antibiotics are given. Patients with an open fracture will be treated according to the local protocol. Patients with a Gustillo grade 1 or 2 open fracture are treated with cefazolin 2 g i.v. three times a day (TID) for 24 h. Patients with an open fractures classified Gustillo 3 receive amoxicillin/clavulanic acid 2.2 g i.v. TID for 72 h [10].

The postoperative pin-site care includes the daily inspection of pin-entry sites, disinfection with Betadine©, followed by a dry-gauze dressing by the nursing staff during inpatient stay. In the further course this is done either by giving outpatient wound care, the family physician, or, in cases of good compliance, the patient.

**Fig. 2:**
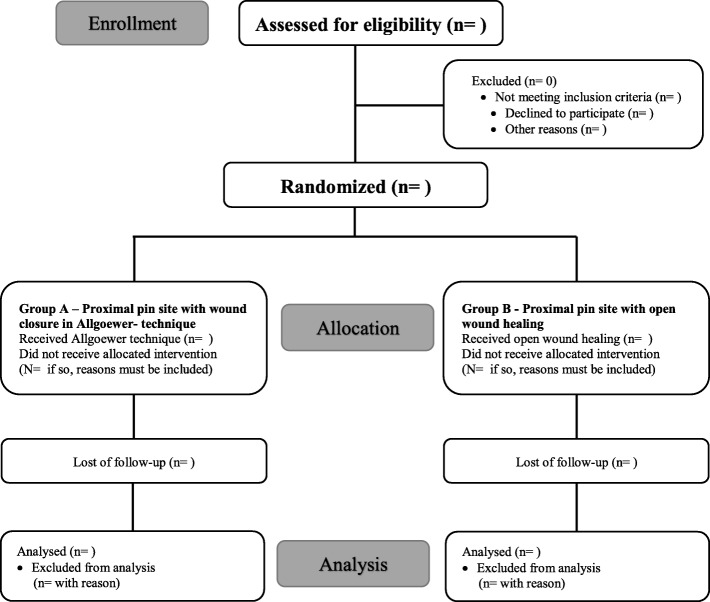
Study Enrollement

**Fig. 3:**
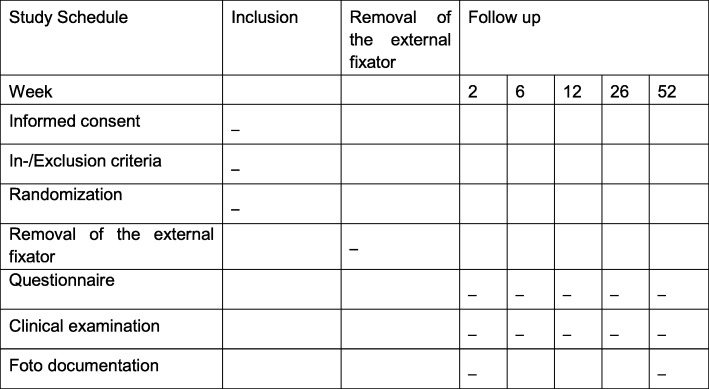
Study Schedule

A photo documentation of the pin sites is made 2 and 52 weeks postoperatively. All patients are clinically followed up regularly at 2, 6, 12, 26 and 52 weeks. The chosen therapy for the examining physician is blinded at the 6-week and 52-week visits. A flowchart is shown in Fig. 2 and the study schedule in Fig. 3.

### Statistical methods

For the primary study objective, it should be shown in a non-inferiority approach that the rate of postoperative wound infection (within 12 weeks of removal of the external fixator) is not significantly greater following simple wound closure of the pin-entry sites than following open secondary wound healing. The non-inferiority limit for this proof is 10%. The primary study objective to be confirmed is achieved when the upper limit of the 95% confidence interval (two-sided) for the difference in infection rates (simple wound closure – open-wound healing) does not exceed the non-inferiority limit of 10%. Similarly, wound infection rates will also be evaluated at the other assessment time points. Furthermore, also for the secondary parameters, the rates of wound healing and the rates of revision surgeries and antibiotic therapies, the proportions per treatment group and time point will be calculated in the same manner as for the primary parameter, and 95% confidence intervals will be presented for the difference in proportions between treatment groups. For the secondary parameters, a comparison with a non-inferiority limit is no longer the main focus. The wound healing rate, for example, is more about being able to possibly deduce from the pattern of proportions an earlier onset of the healing process after simple wound closure. All other parameters will be evaluated purely descriptively.

### Sample size and determination

Seventy patients, resulting in up to 234 pin-entry points, are included in the study. For each patient, up to four individual wounds are treated alternately with simple wound closure or by open-wound healing; the treatment of the proximal pin-entry site per patient is determined by a randomization scheme (1:1). For secondary wound healing, an infection rate of 5% is assumed (within 12 weeks postoperatively), as well as an infection rate of 5% for primary wound healing. Assuming that the infection probabilities of the individual wounds (even within the same patient) are independent, at least 156 evaluable wounds are needed to maintain a non-inferiority limit of 10% for the difference in infection rates with a power of 80%. Inclusion of 70 patients is expected to achieve the required number of wounds (even if individual patients contribute less than four evaluable wounds). With a possible dropout rate of approximately 25% we will generously include 234 pin sites, which equates to around 70 patients in total.

Based on retrospective analyses it is expected that about 50 patients are being treated with an external fixator annually at our hospital. Therefore, the inclusion period will be from January 2019 to the middle of 2020, with an estimated end of this trial 1 year later after the follow-up has been completed.

### Ethical approval

The sponsor, the investigator and Swiss Ethics Board have approved the trial’s protocol version 2, dated 30 October 2018. This trial will be conducted according to the ethical protocol and the current version of the World Medical Association Declaration of Helsinki, the International Conference on Harmonization – Good Clinical Practice (ICH GCP) guidelines and the standards of the International Organization for Standardization (ISO) 14155, applied to the local legal requirements.

### Methods of minimizing bias

To avoid initial bias, all patients are treated identically. Therefore, and to obtain an equal patient population or groups regarding preexisting conditions, health status and trauma condition, all pin sites of the patients are treated at the time of removal of the external fixator alternately by means of primary wound closure and secondary open-wound healing. Randomization only applies to the treatment of the proximal pin site: closed by the Allgoewer single-button technique or left open for secondary open-wound healing, while the rest of the pin sites are treated alternately. This minimizes bias. Included are all pin sites, except those that affect the calcaneus, due to the low mobilizability of the skin and thus a lack of tension-free wound closure. Regarding the back of the hand and foot, a medially located pin site is considered to be the proximal pin site.

## Discussion

To date, there remains a lack of evidence concerning the optimal treatment for pin-site care [5, 11, 12]. The peri- and postoperative management of the pin sites shows a high variability [1, 8]. It is still hard to find a uniform standard that describes how to deal with the pin sites (after application and removal of the fixator extern). There is no consent in preventing pin-site infections, which is reflected in the many hospitals which have different postoperative pin-site care protocols [13]. In one of the most frequently cited publications about pin-site care of an external fixator, a literature review examines the infection rate in terms of pin design, surgical technique, cleaning solutions, frequency of pin-site cleaning, dressing types, effect of showering, and antibiotic prophylaxis [4–6, 14, 15]. In this paper, the treatment of the pin sites after removal of the external fixator is not considered in detail so it is unclear what method leads to a reduction of infections and wound-healing problems. In the authors’ department, after removal of the external fixator, the pin sites are routinely treated by primary wound closure. However, a recently published international survey showed that the majority of surgeons treated the pin site by secondary wound healing [15]. In a review paper, Kazmers et al. discussed different influencing factors for infections of the pin site. Therefore, it is unknown whether the pin design, the surgical technique, different disinfection solutions, the frequency of pin-site cleaning, the dressing type or the choice of antibiotics is important for pin-site infections [14]. In order to address the postoperative management of the pin site, this prospective, randomized controlled trial has been designed. This trial should determine whether the pin sites should be left open or can safely be closed after removal of the external fixator with respect to the occurrence of postoperative wound infection.

This study has some limitations which should be acknowledged. Firstly, this is a single-center study. Although this might make the results less generalizable, single-center studies tend to have more complete data and loss loss-to-follow-up, thus improving the data quality. Secondly, although the study population size is sufficient for detecting differences in primary outcome, it is not large enough for in-depth subgroup analysis.

## Trial status

The Institutional Review Board has approved the study and patient enrollment started in January 2019. In the first 8 months, 51 patients currently with 161 pin sites could be recruited. At the moment, 24 ankle joints, 18 wrists joints, 3 knee joints, 3 elbows and 3 femora with acute trauma were temporarily treated with external fixation. Eleven patients were excluded. To date, no patient showed signs of pin-site infections. Based on our power analysis at enrollment, the last patient is expected in mid-2020. Final follow-up will be finished 1 year later.

## Availability of data and material

The datasets during and/or analyzed during the current study are available from the corresponding author on reasonable request.

## Supplementary information

**Additional file 1.** Standard Protocol Items: Recommendations for Interventional Trials (SPIRIT) 2013 Checklist.

## Data Availability

The datasets used and/or analyzed during the current study are available from the corresponding author on reasonable request.

## Abbreviations

CDC: Center for Disease Control and Prevention
i.v.: Intravenously
ICH GCP Guidelines: International Conference on Harmonization – Good Clinical Practice
ISO: International Organization for Standardization
TID: Three times a day
